# Obesity Is Positively Associated and Alcohol Intake Is Negatively Associated with Nephrolithiasis

**DOI:** 10.3390/nu14194122

**Published:** 2022-10-04

**Authors:** So Young Kim, Dae Myoung Yoo, Woo Jin Bang, Hyo Geun Choi

**Affiliations:** 1 CHA Bundang Medical Center, Department of Otorhinolaryngology-Head & Neck Surgery, CHA University, Seongnam 13496, Korea; 2 Hallym Data Science Laboratory, Hallym University College of Medicine, Anyang 14068, Korea; 3 Department of Urology, Hallym Sacred Heart Hospital, Hallym University College of Medicine, Anyang 14068, Korea; 4 Department of Otorhinolaryngology-Head & Neck Surgery, Hallym University College of Medicine, Anyang 14068, Korea

**Keywords:** urolithiasis, obesity, smoking, risk factors, case-control studies, epidemiology

## Abstract

The current research investigated the impacts of smoking, alcohol consumption, and obesity on the development of nephrolithiasis. We included ≥40-year-old Koreans from the Korean National Health Insurance Service-Health Screening Cohort. A total of 28,395 nephrolithiasis patients were compared with 113,580 control participants. Previous histories of smoking, alcohol consumption, and obesity were examined before the presence of nephrolithiasis. Conditional logistic regression analysis was performed to estimate the odds ratios (ORs) of smoking, alcohol consumption, and obesity for nephrolithiasis. Further analyses were conducted, according to age, sex, smoking, alcohol consumption, and obesity. The current smoking status was not linked with the presence of nephrolithiasis. Alcohol consumption was linked with a lower likelihood of the presence of nephrolithiasis (adjusted OR (aOR) = 0.89, 95% confidence intervals (CI) = 0.86–0.92, *p* < 0.001). Being obese was associated with a greater likelihood of the presence of nephrolithiasis ((95% CI) = 1.27 (1.22–1.31) < 1.42 (1.37–1.46) < 1.59 (1.47–1.71) for overweight < obese I < obese II). The relation of alcohol consumption and obesity with nephrolithiasis was consistent in the subgroups. The presence of nephrolithiasis was positively linked with obesity and negatively linked with alcohol consumption.

## 1. Introduction

Nephrolithiasis is a common disease, whose incidence has been escalating worldwide [1,2]. The prevalence of nephrolithiasis was expected to be about 13% in North America, 5–9% in Europe, and 1–5% in Asia [3]. The prevalence of nephrolithiasis has been higher in men than women, with a men-to-women ratio of 3:1 [4,5]. The reasons for the rising incidence of nephrolithiasis have been attributed to several lifestyle factors, such as obesity and dietary habits of calcium and fluid intake [2]. In addition, increasing incidences of diabetes and cardiovascular diseases have been mentioned as contributors to the occurrence of nephrolithiasis [2].

Lifestyle factors of obesity and dietary factors have been investigated as modifiable risk factors for nephrolithiasis [6,7,8]. The dietary intake of sodium, meat, and soda was listed as a risk factor for nephrolithiasis [7]. In contrast, the dietary intake of fluid, coffee, and calcium was listed as a protective factor for nephrolithiasis [7]. In line with this, another cohort study listed four modifiable risks for nephrolithiasis, including body mass index (BMI), fluid intake, calcium intake, and sugar sweetened drink, as well as a protective factor of dietary approach to stop hypertension style diet [8]. Smoking and alcohol consumption are other lifestyle factors that potentially impact the risk of nephrolithiasis [6]. Smoking was linked to a 2.06 times greater risk of nephrolithiasis (95% CI = 1.06–4.01) [9]. However, the study population was not sufficiently large to declare the risk of smoking on nephrolithiasis [9]. For alcohol consumption, its impact on nephrolithiasis was not definite in a systematic review [6]. A large study population may be needed to resolve the questions regarding the impacts of a number of lifestyle factors on nephrolithiasis. Moreover, these factors should be considered and analyzed concurrently to clarify their independent risks for nephrolithiasis.

We intended to concurrently estimate the influence of lifestyle factors of obesity, smoking, and alcohol consumption on the occurrence of nephrolithiasis in the adult population, when considering other comorbidities. Compared to previous works, this study has virtues regarding the large number of nephrolithiasis patients and control participants, nationwide representative cohort population, and concurrently analyzed multiple lifestyle factors of smoking, alcohol consumption, and obesity. In addition, to minimize the potential confounding effects, comorbidities and laboratory findings were considered in all analyses. Owing to the large cohort population, the comparison population can be designated randomly and matched for demographic factors with the nephrolithiasis patients to diminish the potential bias from the selection process of the study population.

## 2. Methods

**Figure 1:**
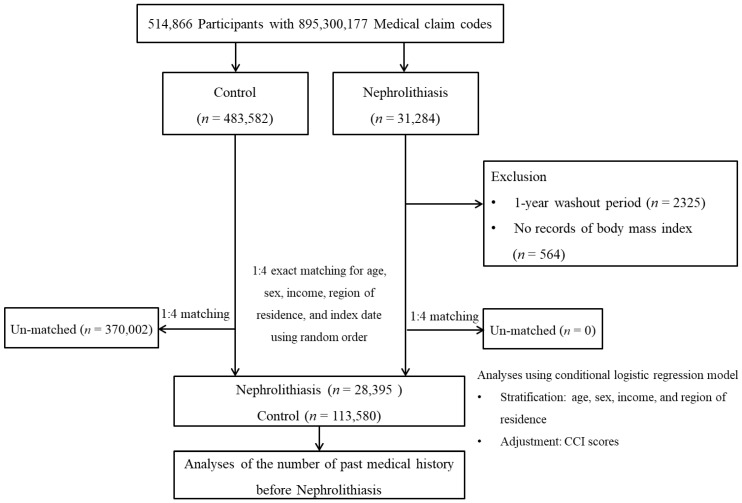
A schematic illustration of the participant selection process used in the present study. Of a total of 514,866 participants, 28,395 nephrolithiasis participants were 1:4 matched with 113,580 control participants for age, sex, income, and region of residence.

Data from the Korean National Health Insurance Service-Health Screening Cohort, from 2002 through 2019, were used [10,11]. The ethics committee of Hallym University (2019-10-023) permitted the current research. The Institutional Review Board exempted the written informed consent. The 31,284 of nephrolithiasis participants were designated from entire cohort population. The 483,582 participants did not have nephrolithiasis. Nephrolithiasis patients identified in 2002 were removed (*n* = 2325). Nephrolithiasis patients who did not have a previous health check-up before nephrolithiasis were removed (*n* = 564). Nephrolithiasis patients were 1:4 matched with control participants for age, sex, income, and region of residence. At last, 28,395 nephrolithiasis patients were 1:4 matched with 113,580 control participants (Figure 1).

Nephrolithiasis (ICD-10 codes: N20) was defined if the participants treated it ≥2 times.

The status of smoking and alcohol intake were classified based on the self-reported survey during health checkup [11]. Based on the body mass index (BMI, kg/m^2^), obesity group was classified [12]. Age groups were divided into 5-year intervals: 40–44, 45–49, 50–54, …, and 85+ years old. Age, income, and region of residence were collected from national health insurance data. The Charlson comorbidity index (CCI) score was used as a covariate in the analyses [13].

To analyze the odds ratios (ORs) of past medical history for nephrolithiasis, conditional logistic regression analysis was conducted. In these analyses, age, sex, income, region, and CCI were adjusted. The standardized difference compared variables between the nephrolithiasis and control groups. For the subgroup analyses using the conditional logistic regression, we stratified participants by their age and sex. Additional subgroup analyses were conducted using unconditional logistic regression, according to obesity, smoking, and alcohol consumption. The *p*-value < 0.05 was set as a statistical significance. SAS version 9.4 (SAS Institute Inc., Cary, NC, USA) was utilized.

## 3. Results

The nephrolithiasis group had a higher rate of obesity than the control group (Table 1, sd = 0.17). A total of 37.8% and 3.8% of the nephrolithiasis group was classified into the obese I and obese II classes, respectively, which were 32.6% and 2.9% of the control group. A total of 17.21% of the nephrolithiasis group and 16.7% of the control group were current smokers (sd = 0.01). The rate of alcohol consumption was lower in the nephrolithiasis group than in the control group (35.06% vs. 37.3%, sd = 0.05).

Smoking, alcohol consumption, and obesity were examined for their association with the occurrence of nephrolithiasis (Table 2). Smoking was related to 1.04 times higher odds for nephrolithiasis in the crude model (95% CI = 1.01–1.08, *p* = 0.023). However, the association of smoking with nephrolithiasis was not maintained in the adjusted model (adjusted OR (aOR) = 1.03, 95% CI = 1.00–1.07, *p* = 0.102. Alcohol consumption was linked with lower odds for nephrolithiasis (aOR = 0.89, 95% CI = 0.86–0.92, *p* < 0.001). On the other hand, obesity was associated with greater odds for nephrolithiasis with dose-response relations (aOR (95% CI) = 1.27 (1.22–1.31) < 1.42 (1.37–1.46) < 1.59 (1.47–1.71) for overweight < obese I < obese II).

These associations of alcohol consumption and obesity with nephrolithiasis were consistent in most age and sex subgroups, except for women group (Table 3). Women group did not show association between alcohol consumption and nephrolithiasis. The inverse association of alcohol consumption with nephrolithiasis was prominent in the <55-year-old (aOR = 0.82, 95% CI = 0.78–0.87) and male (aOR = 0.86, 95% CI = 0.84–0.89) groups. The positive relationship of obesity with nephrolithiasis was noticeable in the ≥55-year-old (aOR = 1.46, 95% CI = 1.40–1.52 for obese I) and male (aOR = 1.56, 95% CI = 1.50–1.63) groups.

Other subgroup analyses according to smoking and alcohol consumption demonstrated similar relations of alcohol consumption and obesity with nephrolithiasis (Table 4). Alcohol consumption was negatively associated with nephrolithiasis in BMI subgroups of normal weight, overweight, and obese I.

## 4. Discussion

The rate of nephrolithiasis was high in the population with obesity in the adult population. In contrast, frequent alcohol consumption was linked with a low rate of nephrolithiasis. The current study enlarged the previous knowledge on the impact of lifestyle factors on the occurrence of nephrolithiasis by analyzing lifestyle factors in a large cohort population. Although previous works demonstrated the relation of smoking and alcohol consumption with the occurrence of nephrolithiasis, the study population of each study was limited and considered only several number of potential confounders [6,14].

Obesity was associated with a greater risk of nephrolithiasis in the current research. In addition, the odds for nephrolithiasis showed a dose-response association with the severity of obesity. A few prior studies have described the risk of nephrolithiasis associated with obesity. In a meta-analysis, the obese population was likely to experience nephrolithiasis, with a pooled relative risk of approximately 1.39 (95% CI = 1.27–1.52) [7]. In a young adult population, obesity with a BMI of 30 or above was linked with 1.7 times (range 1.4–2.1) higher odds for nephrolithiasis [15]. Obesity can mediate the occurrence nephrolithiasis by changing the urinary pH and excreted components [16]. Several previous studies suggested reduced urine pH in obese populations [17,18]. The low urine pH associated with obesity can be explained by insulin resistance and fatty acids in the obese population [19]. Moreover, obese patients demonstrated an elevated urine composition of sodium, uric acid, sulfate, and phosphate [20]. In summary, altered urine contents with acidic features may increase vulnerability to nephrolithiasis in obese patients.

In the current results, alcohol consumption was linked with a low risk of nephrolithiasis. Some prior studies also reported a reduced risk of nephrolithiasis in patients who consumed alcohol [14]. In a meta-analysis, the pooled OR of nephrolithiasis for alcohol consumption was as low as 0.683 (95% CI = 0.577–0.808) [14]. In addition, the protective effect of alcohol consumption on nephrolithiasis showed a dose-dependent association, with a 10% decrease in the rate of nephrolithiasis for a 10 g/day of alcohol consumption [14]. However, there was no protective effect of alcohol consumption on nephrolithiasis when it was excessively consumed [21]. Drinking pure alcohol up to 59.9 g/day was associated with a lower risk of nephrolithiasis than not drinking alcohol (hazard ratio = 0.79, 95% CI = 0.72–0.87), while more alcohol consumption did not lower the risk of nephrolithiasis [21]. Because excessive alcohol consumption can be hazardous, due to the metabolites of ethanol, such as acetaldehyde, precious management should be supplemented for alcohol consumption [22]. Excessive alcohol consumption can induce nephrolithiasis by promoting hypercalciuria and hyperoxaluria, which results in calcium oxalate crystal formation [23]. Alcohol was suggested to reduce the risk of nephrolithiasis by diluting metabolites in the blood and urine [14]. In addition, the diuretic effect of alcohol can promote voiding, which diminishes the risk of nephrolithiasis [24].

The current research used a large, nationwide cohort population, which enhanced the statistical power. Due to the sufficient population, control participants can be randomly chosen and matched for both demographic and socioeconomic factors with nephrolithiasis patients. Because this study was based on nationwide health insurance data and national health check-up data, the diagnosis of diseases and measurements of laboratory data were reliable. However, we cannot specify the severity and recurrence of nephrolithiasis in the current study. In addition, for smoking and alcohol consumption, the data were based on self-report questionnaires; in that, recall bias can be possible. For these variables, we can estimate the dose-response relationship in the present study because our cohort did not examine the duration of smoking and alcohol consumption. For obesity, other parameters for obesity, such as waist to hip ratio and body fat, were not considered in this study. The medication histories were not considered in the present cohort. Finally, this is a retrospective case-control study in that causality cannot be inferred from the present data. Future studies with randomized controlled trials with specified diseases and medications can unravel the present limitations.

## 5. Conclusions

The appropriate alcohol consumption did not increase the risk of nephrolithiasis. In contrast, obesity was a positive predictive factor for a higher risk of nephrolithiasis. Clinicians need to consider the possible impacts of alcohol consumption and obesity for prevention and management of nephrolithiasis.

## Figures and Tables

**Table 1:** General characteristics of participants.

Characteristics	Total Participants		
	Nephrolithiasis (*n*, %)	Control (*n*, %)	Standardized Difference
Age (years old)			0.00
40–44	1072 (3.8)	4288 (3.8)	
45–49	3444 (12.1)	13,776 (12.1)	
50–54	5338 (18.8)	21,352 (18.8)	
55–59	6050 (21.3)	24,200 (21.3)	
60–64	4877 (17.2)	19,508 (17.2)	
65–69	3472 (12.2)	13,888 (12.2)	
70–74	2265 (8)	9060 (8)	
75–79	1278 (4.5)	5112 (4.5)	
80–84	463 (1.6)	1852 (1.6)	
85+	136 (0.5)	544 (0.5)	
Sex			0.00
Male	18,260 (64.3)	73,040 (64.3)	
Female	10,135 (35.7)	40,540 (35.7)	
Income			0.00
1 (lowest)	3983 (14)	15,932 (14)	
2	3324 (11.7)	13,296 (11.7)	
3	4338 (15.3)	17,352 (15.3)	
4	6243 (22)	24,972 (22)	
5 (highest)	10,507 (37)	42,028 (37)	
Region of residence			0.00
Urban	12,400 (43.7)	49,600 (43.7)	
Rural	15,995 (56.3)	63,980 (56.3)	
CCI score (score)			0.07
0	16,779 (59.1)	68,877 (60.6)	
1	5150 (18.1)	18,199 (16)	
2	2913 (10.3)	11,003 (9.7)	
3	1550 (5.5)	6373 (5.6)	
≥4	2003 (7.1)	9128 (8)	
Obesity			0.17
<18.5 (underweight)	392 (1.4)	2691 (2.4)	
≥18.5 to <23 (normal)	8016 (28.2)	38,969 (34.3)	
≥23 to <25 (overweight)	8189 (28.8)	31,561 (27.8)	
≥25 to <30 (obese I)	10,728 (37.8)	37,052 (32.6)	
≥30 (obese II)	1070 (3.8)	3307 (2.9)	
Smoking status			0.01
Nonsmoker or Past smoker	23,508 (82.8)	94,636 (83.3)	
Current smoker	4887 (17.21)	18,944 (16.7)	
Alcohol consumption			0.05
<1 time a week	18,440 (64.94)	71,219 (62.7)	
≥1 time a week	9955 (35.06)	42,361 (37.3)	

Abbreviations: CCI, Charlson comorbidity index.

**Table 2:** Crude and adjusted odd ratios (95% confidence intervals) of smoking, alcohol consumption, and obesity for nephrolithiasis.

Characteristics	N of Nephrolithiasis	N of Control	Odd Ratios for Nephrolithiasis (95% Confidence Interval)			
	(Exposure/Total, %)	(Exposure/Total, %)	Crude ^†^	*p*-Value	Adjusted ^†,‡^	*p*-Value
Smoking status	4887/28,395 (17.2)	18,944/113,580 (16.7)	1.04 (1.01–1.08)	0.023 *	1.03 (1.00–1.07)	0.102
Alcohol consumption	9955/28,395 (35.1)	42,361/113,580 (37.3)	0.90 (0.87–0.92)	<0.001 *	0.89 (0.86–0.92)	<0.001 *
Obesity				<0.001 *		<0.001 *
<18.5 (underweight)	392/28,395 (1.4)	2691/113,580 (2.4)	0.70 (0.63–0.78)	<0.001 *	0.70 (0.63–0.78)	<0.001 *
18.5 to 23 (normal)	8016/28,395 (28.2)	38,969/113,580 (34.3)	1.00		1.00	
23 to 25 (overweight)	8189/28,395 (28.8)	31,561/113,580 (27.8)	1.27 (1.22–1.31)	<0.001 *	1.27 (1.22–1.31)	<0.001 *
25 to 30 (obese I)	10,728/28,395 (37.8)	37,052/113,580 (32.6)	1.42 (1.37–1.46)	<0.001 *	1.42 (1.37–1.46)	<0.001 *
30 (obese II)	1070/28,395 (3.8)	3307/113,580 (2.9)	1.58 (1.47–1.70)	<0.001 *	1.59 (1.47–1.71)	<0.001 *

* Conditional logistic regression analysis, significance at *p* < 0.05. ^†^ Stratified model for age, sex, income, and region of residence. ^‡^ Adjusted model for Charlson comorbidity index, obesity, smoking state (current smoker compared to non-smoker or past smoker), and frequency of alcohol consumption (≥1 time a week, compared to <1 time a week).

**Table 3:** Crude and adjusted odd ratios (95% confidence interval) of smoking, alcohol consumption, and obesity for nephrolithiasis in each stratified group, according to age and sex.

	N of Nephrolithiasis	N of Control	ORs of Nephrolithiasis			
	(Exposure/Total, %)	(Exposure/Total, %)	Crude ^†^	*p*-Value	Adjusted ^†,‡^	*p*-Value
**<55 years old (*n* = 49,270)**						
Smoking	1408/9854 (14.3%)	5527/39,416 (14.0%)	1.02 (0.96–1.09)	0.479	1.03 (0.96–1.10)	0.389
Alcohol consumption	3527/9854 (35.8%)	15,592/39,416 (39.6%)	0.83 (0.79–0.87)	<0.001 *	0.82 (0.78–0.87)	<0.001 *
Obesity (BMI, kg/m^2^)				<0.001 *		<0.001 *
<18.5 (underweight)	125/9854 (1.3%)	647/39,416 (1.6%)	0.91 (0.74–1.10)	0.319	0.90 (0.74–1.09)	0.291
≥18.5 to <23 (normal)	2899/9854 (29.4%)	13,589/39,416 (34.5%)	1.00		1.00	
≥23 to <25 (overweight)	2848/9854 (28.9%)	11,237/39,416 (28.5%)	1.19 (1.13–1.26)	<0.001 *	1.20 (1.13–1.27)	<0.001 *
≥25 to <30 (obese I)	3664/9854 (37.2%)	12,883/39,416 (32.7%)	1.34 (1.27–1.42)	<0.001 *	1.34 (1.27–1.42)	<0.001 *
≥30 (obese II)	318/9854 (3.2%)	1060/39,416 (2.7%)	1.41 (1.24–1.61)	<0.001 *	1.42 (1.24–1.62)	<0.001 *
**≥55 year old (*n* = 92,705)**						
Smoking	3479/18,541 (18.1%)	13,417/74,164 (18.8%)	1.05 (1.01–1.10)	0.023 *	1.03 (0.99–1.08)	0.175
Alcohol consumption	6428/18,541 (34.7%)	26,769/74,164 (36.1%)	0.93 (0.90–0.97)	<0.001 *	0.93 (0.89–0.96)	<0.001 *
Obesity (BMI, kg/m^2^)				<0.001 *		<0.001 *
<18.5 (underweight)	267/18,541 (1.4%)	2044/74,164 (2.8%)	0.64 (0.56–0.73)	<0.001 *	0.64 (0.56–0.73)	<0.001 *
≥18.5 to <23 (normal)	5117/18,541 (27.6%)	25,380/74,164 (34.2%)	1.00		1.00	
≥23 to <25 (overweight)	5341/18,541 (28.8%)	20,324/74,164 (27.4%)	1.31 (1.25–1.37)	<0.001 *	1.31 (1.25–1.36)	<0.001 *
≥25 to <30 (obese I)	7064/18,541 (38.1%)	24,169/74,164 (32.6%)	1.46 (1.40–1.52)	<0.001 *	1.46 (1.40–1.52)	<0.001 *
≥30 (obese II)	752/18,541 (4.1%)	2247/74,164 (3.0%)	1.67 (1.53–1.83)	<0.001 *	1.68 (1.54–1.83)	<0.001 *
**Men (*n* = 91,300)**						
Smoking	4801/18,260 (26.3%)	18,653/73,040 (25.5%)	1.04 (1.00–1.08)	0.035 *	1.02 (0.98–1.06)	0.253
Alcohol consumption	8473/18,260 (46.4%)	36,347/73,040 (49.8%)	0.87 (0.85–0.90)	<0.001 *	0.86 (0.84–0.89)	<0.001 *
Obesity (BMI, kg/m^2^)				<0.001 *		<0.001 *
<18.5 (underweight)	198/18,260 (1.1%)	1771/73,040 (2.4%)	0.57 (0.49–0.66)	<0.001 *	0.57 (0.49–0.66)	<0.001 *
≥18.5 to <23 (normal)	4732/18,260 (25.9%)	24,435/73,040 (33.5%)	1.00		1.00	
≥23 to <25 (overweight)	5457/18,260 (29.9%)	20,854/73,040 (28.6%)	1.36 (1.30–1.42)	<0.001 *	1.36 (1.30–1.42)	<0.001 *
≥25 to <30 (obese I)	7309/18,260 (40.0%)	24,321/73,040 (33.3%)	1.56 (1.50–1.63)	<0.001 *	1.56 (1.50–1.63)	<0.001 *
≥30 (obese II)	564/18,260 (3.1%)	1659/73,040 (2.3%)	1.77 (1.60–1.95)	<0.001 *	1.78 (1.61–1.97)	<0.001 *
**Women (*n* = 50,675)**						
Smoking	86/10,135 (0.9%)	291/40,540 (0.7%)	1.18 (0.93–1.51)	0.171	1.18 (0.93–1.51)	0.177
Alcohol consumption	1482/10,135 (14.6%)	6014/40,540 (14.8%)	0.98 (0.92–1.05)	0.589	0.99 (0.93–1.05)	0.695
Obesity (BMI, kg/m^2^)				<0.001 *		<0.001 *
<18.5 (underweight)	194/10,135 (1.9%)	920/40,540 (2.3%)	0.93 (0.80–1.09)	0.393	0.93 (0.79–1.09)	0.385
≥18.5 to <23 (normal)	3284/10,135 (32.4%)	14,534/40,540 (35.9%)	1.00		1.00	
≥23 to <25 (overweight)	2732/10,135 (27.0%)	10,707/40,540 (26.4%)	1.13 (1.07–1.20)	<0.001 *	1.13 (1.07–1.20)	<0.001 *
≥25 to <30 (obese I)	3419/10,135 (33.7%)	12,731/40,540 (31.4%)	1.19 (1.13–1.26)	<0.001 *	1.19 (1.13–1.26)	<0.001 *
≥30 (obese II)	506/10,135 (5.0%)	1648/40,540 (4.1%)	1.36 (1.23–1.52)	<0.001 *	1.36 (1.22–1.51)	<0.001 *

* Conditional logistic regression analysis, significance at *p* < 0.05. ^†^ Stratified model for age, sex, income, and region of residence. ^‡^ Adjusted model for Charlson comorbidity index, obesity, smoking state (current smoker compared to non-smoker or past smoker), and frequency of alcohol consumption (≥1 time a week compared to <1 time a week).

**Table 4:** Crude and adjusted odd ratios (95% confidence interval) of smoking, alcohol consumption, and obesity for nephrolithiasis in each group.

Characteristics	N of Nephrolithiasis	N of Control	ORs of Nephrolithiasis			
	(Exposure/Total, %)	(Exposure/Total, %)	Crude	*p*-Value	Adjusted ^†^	*p*-Value
**Non or past smoker (*n* = 118,144)**						
Alcohol consumption	7332/23,508 (31.2%)	31,732/94,636 (33.5%)	0.90 (0.87–0.93)	<0.001 *	0.89 (0.86–0.92)	<0.001 *
Obesity (BMI, kg/m^2^)				<0.001 *		<0.001 *
<18.5 (underweight)	358/23,508 (1.5%)	2362/94,636 (2.5%)	0.73 (0.66–0.83)	<0.001 *	0.73 (0.65–0.82)	<0.001 *
≥18.5 to <23 (normal)	6866/23,508 (29.2%)	33,347/94,636 (35.2%)	1.00		1.00	
≥23 to <25 (overweight)	6728/23,508 (28.6%)	25,941/94,636 (27.4%)	1.26 (1.21–1.31)	<0.001 *	1.26 (1.22–1.31)	<0.001 *
≥25 to <30 (obese I)	8650/23,508 (36.8%)	30,131/94,636 (31.8%)	1.39 (1.35–1.44)	<0.001 *	1.40 (1.35–1.45)	<0.001 *
≥30 (obese II)	906/23,508 (3.9%)	2855/94,636 (3.0%)	1.55 (1.42–1.67)	<0.001 *	1.55 (1.43–1.68)	<0.001 *
**Current smoker (*n* = 23,831)**						
Alcohol consumption	2623/4887 (53.7%)	10,629/189,44 (56.1%)	0.90 (0.85–0.96)	0.001 *	0.89 (0.84–0.95)	<0.001 *
Obesity (BMI, kg/m^2^)				<0.001 *		<0.001 *
<18.5 (underweight)	34/4887 (0.7%)	329/189,44 (1.7%)	0.51 (0.35–0.72)	<0.001 *	0.50 (0.35–0.72)	<0.001 *
≥18.5 to <23 (normal)	1150/4887 (23.5%)	5622/189,44 (29.7%)	1.00		1.00	
≥3 to <25 (overweight)	1461/4887 (29.9%)	5620/189,44 (29.7%)	1.27 (1.17–1.39)	<0.001 *	1.28 (1.18–1.40)	<0.001 *
≥25 to <30 (obese I)	2078/4887 (42.5%)	6921/189,44 (36.5%)	1.47 (1.36–1.59)	<0.001 *	1.48 (1.37–1.61)	<0.001 *
≥30 (obese II)	164/4887 (3.4%)	452/189,44 (2.4%)	1.77 (1.47–2.14)	<0.001 *	1.79 (1.48–2.16)	<0.001 *
**Consuming alcohol < 1 time a week (*n* = 89,659)**						
Smoking	2264/18,440 (12.3%)	8315/71,219 (11.7%)	1.06 (1.01–1.11)	0.024 *	1.02 (0.97–1.08)	0.458
Obesity (BMI, kg/m^2^)				<0.001 *		<0.001 *
<18.5 (underweight)	287/18,440 (1.6%)	1799/71,219 (2.5%)	0.73 (0.65–0.83)	<0.001 *	0.74 (0.65–0.84)	<0.001 *
≥18.5 to <23 (normal)	5418/18,440 (29.4%)	24,928/71,219 (35.0%)	1.00		1.00	
≥23 to <25 (overweight)	5254/18,440 (28.5%)	19,457/71,219 (27.3%)	1.24 (1.19–1.30)	<0.001 *	1.24 (1.19–1.30)	<0.001 *
≥25 to <30 (obese I)	6750/18,440 (36.6%)	22,835/71,219 (32.1%)	1.36 (1.31–1.42)	<0.001 *	1.36 (1.31–1.42)	<0.001 *
≥30 (obese II)	731/18,440 (4.0%)	2200/71,219 (3.1%)	1.53 (1.40–1.67)	<0.001 *	1.55 (1.42–1.69)	<0.001 *
**Consuming alcohol ≥ 1 time a week (*n* =52,316)**						
Smoking	2623/9955 (26.4%)	10,629/42,361 (25.1%)	1.07 (1.02–1.12)	0.010 *	1.04 (0.99–1.10)	0.139
Obesity (BMI, kg/m^2^)				<0.001 *		<0.001 *
<18.5 (underweight)	105/9955 (1.1%)	892/42,361 (2.1%)	0.64 (0.52–0.78)	<0.001 *	0.63 (0.51–0.77)	<0.001 *
≥18.5 to <23 (normal)	2598/9955 (26.1%)	14,041/42,361 (33.2%)	1.00		1.00	
≥23 to <25 (overweight)	2935/9955 (29.5%)	12,104/42,361 (28.6%)	1.31 (1.24–1.39)	<0.001 *	1.32 (1.24–1.40)	<0.001 *
≥25 to <30 (obese I)	3978/9955 (40.0%)	14,217/42,361 (33.6%)	1.51 (1.43–1.60)	<0.001 *	1.52 (1.44–1.61)	<0.001 *
≥30 (obese II)	339/9955 (3.4%)	1107/42,361 (2.6%)	1.66 (1.46–1.88)	<0.001 *	1.66 (1.46–1.89)	<0.001 *
**Underweight (BMI < 18.5, *n* = 3083)**						
Smoking	34/392 (8.7%)	329/2691 (12.2%)	0.68 (0.47–0.99)	0.043 *	0.96 (0.65–1.41)	0.825
Alcohol consumption	105/392 (26.8%)	892/2691 (33.2%)	0.74 (0.58–0.94)	0.012 *	0.89 (0.69–1.15)	0.377
**Normal weight (BMI ≥ 18.5 to <23, *n* = 46,985)**						
Smoking	1150/8016 (14.4%)	5622/38,969 (14.4%)	0.99 (0.93–1.06)	0.852	1.08 (1.00–1.16)	0.045 *
Alcohol consumption	2598/8016 (32.4%)	14,041/38,969 (36.0%)	0.85 (0.81–0.90)	<0.001 *	0.88 (0.83–0.93)	<0.001 *
**Overweight (BMI ≥ 23 to <25, *n* = 39,750)**						
Smoking	1461/8189 (17.8%)	5620/31,561 (17.8%)	1.00 (0.94–1.07)	0.943	1.00 (0.93–1.07)	0.992
Alcohol consumption	2935/8189 (35.8%)	12,104/31,561 (38.4%)	0.90 (0.85–0.95)	<0.001 *	0.88 (0.83–0.93)	<0.001 *
**Obese I (BMI ≥ 25 to <30, *n* = 47,780)**						
Smoking	2078/10,728 (19.4%)	6921/37,052(18.7%)	1.05 (0.99–1.11)	0.104	1.01 (0.95–1.07)	0.718
Alcohol consumption	3978/10,728 (37.1%)	14,217/37,052(38.4%)	0.95 (0.91–0.99)	<0.015 *	0.90 (0.86–0.95)	<0.001 *
**Obese II (BMI ≥ 30, *n* = 4377)**						
Smoking	164/1070 (15.3%)	452/3307 (13.7%)	1.14 (0.94–1.39)	0.175	1.09 (0.88–1.35)	0.415
Alcohol consumption	339/1070 (31.7%)	1107/3307 (33.5%)	0.92 (0.80–1.07)	0.279	0.86 (0.74–1.01)	0.073

* Unconditional logistic regression analysis, significance at *p* < 0.05. ^†^ Adjusted model for age, sex, income, region of residence, Charlson comorbidity index, obesity, smoking state (current smoker compared to nonsmoker or past smoker), and frequency of alcohol consumption (≥1 time a week compared to <1 time a week).

## Data Availability

Releasing of the data by the researcher is not legally permitted. All data are available from the database of the Korea Centers for Disease Control and Prevention. The Korea Centers for Disease Control and Prevention allows data access, at a particular cost, for any researcher who promises to follow the research ethics. The data of this article can be downloaded from the website, after agreeing to follow the research ethics.
